# Interaction of Dietary Fatty Acids with Tumour Necrosis Factor Family Cytokines during Colon Inflammation and Cancer

**DOI:** 10.1155/2014/848632

**Published:** 2014-04-30

**Authors:** Jiřina Hofmanová, Nicol Straková, Alena Hyršlová Vaculová, Zuzana Tylichová, Barbora Šafaříková, Belma Skender, Alois Kozubík

**Affiliations:** ^1^Department of Cytokinetics, Institute of Biophysics, Academy of Sciences of the Czech Republic, v.v.i., Královopolská 135, 612 65 Brno, Czech Republic; ^2^Institute of Experimental Biology, Department of Animal Physiology and Immunology, Faculty of Science, Masaryk University, Kotlářská 2, 611 37 Brno, Czech Republic

## Abstract

Intestinal homeostasis is precisely regulated by a number of endogenous regulatory molecules but significantly influenced by dietary compounds. Malfunction of this system may result in chronic inflammation and cancer. Dietary essential n-3 polyunsaturated fatty acids (PUFAs) and short-chain fatty acid butyrate produced from fibre display anti-inflammatory and anticancer activities. Both compounds were shown to modulate the production and activities of TNF family cytokines. Cytokines from the TNF family (TNF-**α**, TRAIL, and FasL) have potent inflammatory activities and can also regulate apoptosis, which plays an important role in cancer development. The results of our own research showed enhancement of apoptosis in colon cancer cells by a combination of either docosahexaenoic acid (DHA) or butyrate with TNF family cytokines, especially by promotion of the mitochondrial apoptotic pathway and modulation of NF**κ**B activity. This review is focused mainly on the interaction of dietary PUFAs and butyrate with these cytokines during colon inflammation and cancer development. We summarised recent knowledge about the cellular and molecular mechanisms involved in such effects and outcomes for intestinal cell behaviour and pathologies. Finally, the possible application for the prevention and therapy of colon inflammation and cancer is also outlined.

## 1. Introduction

Immune homeostasis in the intestine is tightly regulated by crosstalk between commensal bacteria, mucosal immune cells, and intestinal epithelial cells. These cells migrate from their place of origin at the crypt base to the villus (small intestine) or crypt (large bowel) tip, from where they are shed into the lumen. Dynamic balance between cell production at the base and cell death at the surface of the colonic crypts is precisely regulated by a number of physiological endogenous factors. In addition to hormones and cytokines, specific signalling through Wnt, Notch, and BMP pathways are essential for intestinal development and homeostasis [1]. Irregularities of such regulation might cause different pathologies including colon cancer. Recently, various types of cell death and their crosstalk were identified in the intestinal epithelium. In addition to apoptosis, autophagy and necroptosis were described as other modes of programmed colon cell death. Excessive cell death has been associated with chronic inflammation in patients with Crohn's disease and ulcerative colitis (UC) and dysregulation of cell death also plays essential role in colorectal cancer (CRC) [2].

The pathogenesis of CRC is a long and multifactorial process which involves not only mutations in specific oncogenes and tumour suppressor genes, but also alterations in gene expression which are induced by epigenetic and nongenotoxic mechanisms [3, 4]. Disturbed regulation of apoptosis and loss of sensitivity to apoptosis-inducing factors are some of the key mechanisms in CRC development [5, 6]. Further, autophagy, process of cellular self-digestion, has been demonstrated to promote cancer cell survival and drug resistance, but on the other hand it may function as a tumour suppressor mechanism [7]. Moreover, an association between inflammation and cancer has been suggested for a long time [8]. Patients with inflammatory bowel disease (IBD) have a significantly increased risk of developing malignancy in the colon [9, 10]. An important role is especially played by increased production of various endogenous inflammatory molecules and malfunction of the immune system. However, some types of these molecules, such as tumour necrosis factor (TNF) family cytokines, may play a dual role. Their overproduction supports inflammation, but they can also induce death receptor-mediated apoptosis in certain cell types [11].

Nowadays it is clear that, in addition to endogenous regulators influencing cell and tissue homeostasis, lifestyle factors (mainly smoking, composition of the diet, and physical activity) play a role in the aetiology of colon inflammation and cancer [12, 13]. Among them, various types of fatty acids originating from dietary fat and fibre are being investigated to clarify the role and possible mechanisms by which they may influence colonic health [14–17].

The results of some studies including our own also suppose that there is mutual interaction between physiological regulators of cell behaviour and dietary lipids, which may positively or negatively influence the maintenance of intestinal tissue homeostasis. In the intestine, signals from nutritional compounds and endogenous factors, which play a role in inflammatory response and regulate cell growth, differentiation, and apoptosis, are integrated within the cell and may have a substantial impact determining the final phenotype, metabolism, and kinetics of epithelial cell population. Moreover, an altered sensitivity and response of transformed cells to these signals play the role during colon carcinogenesis.

In this review we focused on the role and mechanisms of action of dietary essential polyunsaturated fatty acids (PUFAs), short-chain fatty acid (SCFA) butyrate from fibre, and their interaction with TNF family cytokines during colon inflammation and cancer.

## 2. Chronic Inflammation Drives Cancer Development

Chronic inflammation is considered as one of the key mechanisms promoting and accelerating cancer development. This process mainly involves continuous activity of various cytokines, chemokines, production of reactive oxygen and nitrogen species (RONS), activity of certain enzymes, and activation of specific signalling pathways and transcription factors [18].

The inflammation present in the tumour microenvironment is characterised by leukocyte infiltration. These cells produce a variety of cytotoxic mediators such as RONS, proteases, matrix metalloproteinases, TNF-*α*, interleukins (IL), interferons (IFN), and enzymes such as cyclooxygenase-2 (COX-2), 5-lipoxygenase (5-LOX), and phospholipase A2 (PLA2) responsible for eicosanoid formation. Such mediators activate specific transcription factors: nuclear factor *κ*B (NF-*κ*B), AP-1 or hypoxia-inducing factor 1*α* (HIF-1), and signal transducers like Janus kinase/Signal transducers and activators of transcription 3 (JAK/STAT3) [19]. These conditions can induce genetic and epigenetic changes including various types of mutations, chromosomal aberrations, methylation of various tumour-related genes, and modulated expression of microRNAs. These events work in concert to alter the important pathways involved in the normal cellular function and hence accelerate inflammation-associated cancer development [20].

### 2.1. Intestinal Inflammation and Cancer

In patients with IBD, chronic inflammation represents a major risk factor for the development of CRC [21]. This process leads to a disruption of the epithelial barrier and the formation of epithelial ulceration [22]. It permits easy access for the luminal microbiota and dietary antigens to cells resident in the lamina propria and stimulates further pathological immune cell responses [23]. However, the mechanisms underlying this neoplastic transformation are not fully understood. Studies in experimental models of CRC suggest that inflammatory cell-derived cytokines either directly or indirectly stimulate the uncontrolled growth of cancer cells [24].

Despite the differences between the molecular abnormalities found in colitis-associated dysplasia in comparison with sporadic CRC, there are many similarities (dysplasia-cancer sequence, similar frequencies of major chromosomal abnormalities, microsatellite instability, and similar glycosylation changes) that make it reasonable to suggest that also sporadic colon cancer might be largely secondary to inflammation. The fact that regular use of nonsteroidal anti-inflammatory drugs (NSAIDs) can lower the mortality and result in regression of adenomas in familial adenomatous polyposis (FAP) patients with mutation in the adenomatous polyposis coli (APC) gene brings further evidence of the role of inflammation in CRC [25]. However, this process may function as a double-edged sword. Under specific inflammatory conditions, immune cells can boost an antitumour immune response with the downstream effect of eliminating dysplastic and cancerous cells. Thus, inflammation can play both a beneficial and a detrimental role in colon carcinogenesis [26, 27]. Since understanding of the definition and pathogenesis of CRC in IBD is crucial to optimise patient management, further investigation is necessary.

## 3. The Role of Cytokines in Colon Inflammation and Cancer

A variety of immune mediated bowel disorders, including celiac disease, Crohn's disease, and UC, are characterized by accelerated epithelial cell turnover and cell death leading to altered crypt morphology. These changes are mediated by the cytokines released from infiltrating inflammatory cells and enterocytes in paracrine or autocrine fashion, respectively. Similarly, various types of cytokines and chemokines, which can be produced by tumour cells themselves or by the cells in the tumour microenvironment, play an important role in colon cancer development. Using a mouse model of UC, TNF-*α* has been identified as a crucial mediator of the initiation and progression of colitis-associated CRC [28]. Proinflammatory molecules promote the growth of tumour cells, perturb their differentiation, and support the survival of cancer cells [23]. TNF-*α*, interleukins (IL-6 and IL-1*β*), or transcription factors (NF-*κ*B and STATs) that are required for signalling by these cytokines are indeed emerging as potential targets for anticancer therapy. Members of the IL-12 family have been implicated in the pathogenesis of colitis, and IL-23 seems to be involved in inflammation-associated carcinogenesis [29]. The described mediators and activated signalling pathways generally delay or suppress the apoptosis of intestinal cells and modulate angiogenesis and drug-metabolising enzyme induction [30]. It was also reported that the colonic mucosa of UC patients displayed an increased T-cell infiltration. Moreover, the disbalance between CD4+ T-helper subsets (Th1 and Th2) producing specific inflammatory cytokines in the intestine and their resistance to apoptosis contributes to chronic mucosal inflammation [31]. Experiments using the Apc (Min/+) mouse model confirmed the association between intestinal cytokines and tumorigenesis. The overall polyp number and abundance of large polyps significantly correlated with inflammatory cytokine (IL-1, IL-6, and TNF-*α*) response. Inflammatory mediators may thus serve as important biomarkers for CRC progression [32]. Next, we focused on the role of TNF family cytokines.

### 3.1. Dual Role of TNF Family Cytokines

The TNF superfamily is composed of 19 ligands and 29 receptors, which play a highly diverse role in the body. This implies that at least some of the ligands have to interact with more than one receptor. All ligands and their receptors are well described in a recent work of Aggarwal et al. [33]. Without exception, the TNF superfamily exhibits a proinflammatory activity; in part through activation of the transcription factor NF-*κ*B. However, these cytokines are involved in the regulation of not only inflammation but also cellular proliferation, apoptosis, and morphogenesis [11, 34]. Cell signalling is mediated through the interaction between transmembrane receptors and either soluble or transmembrane ligands. TNF-*α*, TRAIL (tumour necrosis factor-related apoptosis-inducing ligand), or Fas ligand (FasL), are expressed in both forms. Members of the TNF superfamily can be classified into those groups with or without an intracellular death domain (DD), and they have been identified as important participants in the regulation of apoptosis [35]. The death domain, a region of approximately 45 amino acids long, plays a crucial role in transmitting the death signals from the cell surface to the intracellular pathways [33, 36]. The cytokines TNF-*α*, FasL, and TRAIL induce apoptosis by binding to their receptors TNFR1/2, Fas, and DR4/DR5, respectively, which possess intracellular DDs recruiting certain adaptor molecules to form the death-inducing signalling complex (DISC) consisting of Fas-associated DD protein (FADD) and procaspase-8 [37]. The activation of caspase-8 at the DISC can be potently regulated by cellular FLICE-like inhibitory protein (cFLIP). In the so-called type I cells, efficient caspase-8 activation is followed by a direct cleavage of effector caspases (extrinsic apoptotic pathway), while type II cells mostly use caspase-8 to cleave the Bid protein, which is responsible for translocation of the apoptotic signal to mitochondria (intrinsic apoptotic pathway) [38]. Changes in mitochondria (mitochondrial transition pore opening, decrease of mitochondrial membrane potential, and production of ROS) are associated with the activation of pro- and antiapoptotic proteins of the Bcl-2 family, which play a crucial role in the release of proapoptotic proteins (cytochrome c, Smac/DIABLO) into the cytosol. The subsequent activation of caspase-9 is followed by activation of effector caspases, cleavage of poly ADP-ribose polymerase (PARP), and overall apoptosis. Caspase activity can be effectively modulated by the inhibitor of apoptosis proteins (IAPs) [39].

Recently, another mode of caspase-independent, nonapoptotic programmed cell death induced by stimulating death receptors named necroptosis was detected. This process shares some signalling pathways and molecules with apoptosis and its interaction with autophagy is also suggested. Necroptosis contributes to regulation of immune system and plays a role in cancer development [40]. It is associated with increased expression of receptor-interacting proteins RIP1/RIP3 and erroneous function of FADD and caspase-8 which may be critical for regulation of intestinal homeostasis [2]. Association of necroptosis and intestinal inflammation through specific activation of TNF-*α* synthesis by RIP1 and Akt kinase pathway has been documented [41].

In summary, TNF cytokines may play a dual role in the intestine; they have potent proinflammatory activities, but they also function as regulators of apoptosis associated with cancer development. It seems that cell proliferation, survival, and apoptosis are activated simultaneously by TNF members and the balance in their production and activation significantly determines the fate of the cells and contributes to intestinal homeostasis. Excessive programmed cell death promotes inflammation and, on the other hand, resistance to apoptosis contributes to cancer development. However, molecular mechanisms are not fully understood and may occur at different levels of intracellular signalling pathways.

### 3.2. TNF-*α*

The first member of the TNF superfamily discovered was TNF-*α*, a pleiotropic proinflammatory cytokine [42]. It plays a crucial role in immune and inflammatory processes, and in endotoxic shock [33, 43]. TNF-*α* is synthesised by macrophages and other cells in response to bacterial toxins, inflammatory products, and other invasive stimuli [44]. Its prolonged production is associated with cancer and chronic infections. It has been suggested that a gut with an active injury (e.g. in Crohn's disease or UC) contains an increased number of TNF-*α* secreting cells [45]. The proinflammatory cytokines, such as TNF-*α*, are typical activators of the canonical NF-*κ*B signalling cascade, which is activated in response to injury, infection, inflammation, and other stress conditions [46]. In extracts of colorectal tumour tissues resected from human patients, a higher endogenous TNF-*α* was detected compared to adjacent normal tissue [47]. In addition to its role in inflammation, TNF-*α* can significantly modulate the proliferation, differentiation, and cell death of colonocytes during cancer progression [48].

### 3.3. TRAIL

TRAIL is an interesting candidate for anticancer therapy because of its ability to selectively induce apoptosis in cancer but not normal cells [49]. TRAIL can interact with at least five different receptors. Two of them, DR4 (TRAIL-R1) and DR5 (TRAIL-R2), signal apoptosis, while decoy receptors DcR1 (TRAIL-R3), DcR2 (TRAIL-R4), and osteoprotegerin (TRAIL-R5) are unable to transmit the apoptotic signal [50].

An important part of TRAIL action is constituted by the regulation of intercellular interactions among the cells of the innate and adaptive immune system and the resulting apoptotic response. TRAIL is expressed on macrophages, dendritic cells, T cells, or NK cells in dependence on their stimulation status, being implicated in immunosuppressive, immunoregulatory, and immune-effector functions. Within this system, TRAIL plays an important role in autoimmune disorders, viral and bacterial infections, and immune surveillance of tumours and metastases [51]. Interestingly, a novel way to target and kill colon and prostate cancer cells in the bloodstream has been reported, using leukocytes presenting TRAIL on their surface along with E-selectin receptor [52].

In addition to acting as a potent tumour-selective inducer of apoptosis, TRAIL is also capable of efficiently triggering the nonapoptotic pathways involving extracellular signal-regulated kinases (ERK), c-Jun N-terminal kinases (JNK), p38, phosphoinositide 3-kinase (PI3K)/Akt or NF-*κ*B, which may result in increased proliferation, survival, migration, invasiveness, or inflammation in different normal and/or cancer cell types [53, 54]. Our results showed that specific inhibitors of the ERK pathway efficiently sensitised human colon cancer cells to TRAIL-induced apoptosis, which was mediated via downregulation of Mcl-1 protein. Moreover, inhibition of the PI3K/Akt pathway in colon cancer cells also resulted in sensitisation to TRAIL-induced apoptosis [55, 56]. It has recently been shown that cancer cells surviving the fractional killing by TRAIL exhibit NF-*κ*B-dependent inflammatory phenotypes [57]. Other studies have shown an enhancement of NF-*κ*B signalling in cancer cells resistant to TRAIL-induced apoptosis, which was associated with promotion of tumour metastasis and invasiveness [58].

The expression of TRAIL DRs, and DcRs may significantly change depending on the stage of tumorigenesis or the inflammatory disorders, suggesting their possible functional role in regulating the course of the disease. TRAIL and DR4 were downregulated in enterocytes, and TRAIL was upregulated in mononuclear cells only in IBD but not in the normal colon or appendicitis. This may point to a pathophysiological role of the TRAIL system in IBD [59]. Moreover, DR4 and DR5 were upregulated in colon adenomas and carcinomas compared with adjacent normal epithelial cells [60]. There exist contradictory data showing both a positive and a negative role of the TRAIL/TRAIL-R system in the regulation of cancer cell apoptosis, motility, invasiveness, and metastasis. DR5 has been reported to mediate anoikis (detachment-induced apoptosis) in human colon cancer cells [61]. DR deficiency in mice has been shown to enhance lymph node metastasis of epithelial cancers [62]. On the other hand, the ability of TRAIL and its DRs to promote metastasis in human and mouse CRC has also been demonstrated [63]. This dual role of DRs, and the differences observed in the cell response to TRAIL may depend on tumour type and stage and cell context, or they might be related to activation of the specific kinase pathways. It has been shown by us and others that cell sensitivity to TRAIL-induced apoptosis may be altered significantly during the colon carcinogenesis [64, 65]. Moreover, in some tumour types, the beneficial apoptosis-inducing effects of TRAIL may not be easily separated from the unfavourable nonapoptotic properties when using TRAIL in cancer therapy.

### 3.4. Fas/FasL

The Fas/FasL system is implicated in the aetiology of IBD because the Fas receptor (also called CD95, APO-1/Fas, or TNFRSF6) is highly expressed in the basolateral membrane of intestinal epithelial cells. During this disease, epithelial cell apoptosis is increased [7]. Fas initiates an apoptotic signal to apoptosis-sensitive cells when oligomerised by a natural ligand, FasL, or anti-Fas antibody [66]. FasL is a transmembrane protein that is expressed by lymphocytes, mainly by CD4+ and CD8+ T cells and B cells, after engagement of the Ag-specific T- or B-cell receptor and macrophages and also by NK cells [67]. It has been proposed that only membrane-bound FasL induces Fas-mediated apoptosis, whereas sFasL triggers nonapoptotic signalling pathways [68]. Activation and infiltration of tumour-specific lymphocytes expressing membrane-bound FasL, primarily tumour-specific CD8+, may be essential for Fas function. Recent results indicated that the colonic mucosa from patients with UC harbours more T cells than the normal colon tissue. This observation is consistent with human clinical data and animal-based studies, which showed a critical role of T cells in UC pathogenesis [69]. While Fas is expressed in every colonocyte of the normal colon mucosa, it is downregulated or lost in the majority of colon carcinomas. In contrast to the normal colonic epithelium, many colon carcinoma cell lines are relatively resistant to Fas cross-link [67]. Genomic data indicate that Fas is not focally amplified but significantly deleted across the entire dataset of 3131 tumours, including human CRC [70]. Thus, these data strongly suggest that Fas functions as a tumour suppressor [71, 72]. It was reported that mice with Fas deficiency in the colon tissue are hypersensitive to induced colitis and mice lacking FasL exhibited a more severe and persistent colitis than normal mice [73]. Several studies showed that cFLIP is an inhibitor of Fas signalling, which enhanced the frequency and decreased latency of subcutaneous tumour growth [67, 74]. However, recent data demonstrated that cFlip is required for intestinal tissue homeostasis in mice. This protein controls the level of activation of caspase-8 to promote the survival of intestinal epithelial cells. When cFlip was deleted from the intestinal epithelium, the animals died within a few days from severe tissue destruction, epithelial cell death, and intestinal inflammation. The death of intestinal cells was regulated extrinsically and required the presence of death receptor ligands, such as TNF-*α* and FasL [75]. Fas is a target for the NF-*κ*B transcription factor but a direct interaction between NF-*κ*B and the Fas promoter in human colon carcinoma cells was not identified [76].

Taken together, the ability of TNF family cytokines to induce apoptosis in intestinal epithelial cells seems to be very important. Their sensitivity may be influenced by many other factors and is altered during inflammation and carcinogenesis. Since many cancer cells become resistant to TNF cytokine-mediated killing, much effort has been devoted to find a possibility of overcoming it by combination with other anticancer agents. In addition to chemotherapeutics, the supportive effects of nontoxic dietary compounds seem to be a promising way. In the following parts we summarise recent knowledge about the effects of such compounds in colon inflammation and cancer and their use in combination with TNF family cytokines.

## 4. Dietary Factors and Colon Cancer

It is pointed out that since the Industrial Revolution, humans have fundamentally changed their dietary habits towards increased consumption of animal fat, decreased consumption of antioxidants, and an increased n-6/n-3 PUFA ratio [77]. The imbalance between energy intake and expenditure mainly due to high fat consumption and low fibre intake represents an important factor influencing colon cancer development. Nowadays, it is recognised that excessive lipid uptake in adipocytes leads to hypertrophy and consequently to metabolic dysregulation, hypoxia, inflammation, impaired adipocytokine expression and angiogenesis, insulin resistance, and macrophage recruitment. In obese patients, tumours commonly colocalise with excessive adipose tissue accumulation producing inflammatory mediators, and most of the features of hypertrophic adipose tissue are observed in cancer patients, namely, those with breast and colon cancer [78, 79]. Using the model of Apc (Min/+) mice, an association of high-fat American-type diet with obesity and an increased number of large polyps was demonstrated. On the other hand, it was also shown that calorie restriction and several bioactive food components, such as n-3 PUFA, can inhibit genetically predisposed CRC [80, 81].

### 4.1. The Role of Polyunsaturated Fatty Acids (PUFAs) in Colon Inflammation and Cancer

It has been shown that the quantitative and qualitative content of essential PUFAs in the diet is highly important [82, 83]. These PUFAs cannot be synthesised by mammals and their availability depends on external supply. They are divided into two main types of the n-6 and n-3 series. Precursor linoleic acid (LA, 18:2, n-6) found in many plant oils (soybean, sunflower) is desaturated and elongated to other types, mainly arachidonic acid (AA, 20:4). Alpha-linolenic acid (ALA, 18:3, n-3) is a precursor for eicosapentaenoic (EPA, 20:5) or docosahexaenoic (DHA, 22:6) acids, which are also found in fish or algal oils. Excessive amounts of n-6 PUFAs and a high n-6/n-3 ratio, found in Western diets, may promote the pathogenesis of many diseases such as cardiovascular disease, obesity, diabetes, autoimmune disease, inflammation, and cancer [84–86]. Recently, it was reported that the fatty acid profile of visceral white adipose tissue correlates with inflammatory signatures potentially associated with CRC [87].

Ingestion of PUFAs leads to their distribution to virtually every cell in the body and influences the lipid profile and fatty acid composition of plasma, nuclear, and mitochondrial cell membranes. This consequently affects the membrane structure and fluidity, the functions of membrane-bound proteins, and lipid-mediated signalling [88]. In IBD patients and human colorectal adenomas and adenocarcinomas, altered activities of enzymes metabolising endogenous AA, particularly increased expression and activity of PLA2, and COX-2 accompanied by an overproduction of prostaglandin E2 (PGE2) were detected [89] and have been shown to be responsible for immunosuppression and tumour promotion [31, 90]. Moreover, IBD colon biopsies show a marked increase in both 5-LOX and leukotriene B4 [91]. Both EPA and DHA compete for fatty acid metabolising enzymes with AA and thus are able to inhibit the formation of AA-derived proinflammatory and immunosuppressive eicosanoids [92]. Importantly, EPA and DHA form several own potent anti-inflammatory mediators (e.g., resolvins and protectins) [93]. The effects of PUFAs and their metabolites on various levels of cell organisation and their interaction with other endogenous or exogenous factors can finally significantly influence cell proliferation, differentiation, and apoptosis of various cell types [94, 95].

During the last 25 years, hundreds of papers describing the effects of PUFAs on various types of normal and cancer cells, differences between n-6 and n-3 PUFAs, and proposed mechanisms of their action have been published. This large and complex topic is beyond the scope of this review. However, in spite of much contradiction in the literature [96], it is generally thought that high calorie and fat intake are risk factors especially in colon, breast, or prostate cancer development and that n-6 PUFAs (from plant oils rich in LA) can promote inflammation and carcinogenesis [97]. Supplementation of diet with PUFAs can substantially influence cell physiology and cell kinetics, mostly by the modulation of oxidative metabolism and biosynthesis of PUFA metabolites. The particular species of free radicals affect specific phases of carcinogenesis in different ways [98]. The effects of n-6 PUFAs are primarily mediated by AA-derived eicosanoids. The anti-inflammatory and anticancer effects of NSAIDs, which function as specific inhibitors of AA metabolism, confirmed the significance of eicosanoids in cancer development [99, 100].

On the other hand, there is growing evidence that n-3 PUFAs, namely, DHA and EPA, found in fish and algal oils, exert anti-inflammatory properties, thus suppressing IBD and colon cancer [101–106]. Over the last years, these n-3 PUFA properties were confirmed by experiments with cell cultures and laboratory animals using the introduction of a newly discovered gene encoding n-3 fatty acid desaturase (fat-1), which is not normally present in mammals [107].

Nutritionally induced changes in fatty acid composition may result in an increased sensitivity to chemo- and radiotherapy and decreased undesirable side effects [108–110]. There is also evidence suggesting that n-3 PUFA may uniquely regulate stem cell signalling pathways and the increased sensitivity of colon cancer cells to chemotherapy by upregulation of colonic differentiation markers [111].

There are many papers describing the different mechanisms considered to be responsible for n-3 PUFA effects on cellular and molecular levels [101, 106, 112–114]. In general, there are three major mechanisms suggested to play the main role in n-3 PUFA effects: (1) alteration of cellular membrane phospholipid composition and lipid microdomain functionality, (2) competition for metabolisation enzymes with n-6 PUFAs and thus production of bioactive mediators, eicosanoids, and (3) modulation of intracellular signalling and nuclear receptor activation. These aspects are discussed in a further chapter dealing with the interaction of PUFAs with TNF family cytokines.

### 4.2. Fibre and Short-Chain Fatty Acids (SCFAs) in Colon Inflammation and Cancer

Dietary fibre increases the gastrointestinal tract biomass, changes the composition of gut flora, and may decrease the risk of metabolic disorders like dyslipidaemia, hypercholesterolaemia, and hyperglycaemia and also substantially influence immune-based actions [115]. Its fermentation by intestinal bacteria leads to the generation of SCFAs propionate, acetate, and butyrate. Butyrate, present in the colonic lumen in millimolar concentration, acts as a principal energy source and a survival factor for normal colon cells. In addition, it possesses anti-inflammatory and antineoplastic properties. Butyrate supplementation dramatically reduced proliferation and induced differentiation and/or cell death in colon cancer cells [116]. In addition to the regulation of basic cytokinetic processes, butyrate has also been shown to affect cell adhesion, morphology, invasiveness, metastasis, oxidative metabolism, angiogenesis, activity of different enzymes, gene expression and chromatin modulation, activity/expression of various transcription factors, and signal transduction molecules. These multiple effects in colon cells were reviewed by us previously [117].

The butyrate action is very complex and still not completely understood. Butyrate is a well-known histone deacetylase (HDAC) inhibitor and thereby regulates gene expression and induces sensitisation effects on cytokine action in colon cancer cells [118, 119]. Accompanying these changes butyrate influences the cell response to inflammatory stimuli mainly by the inhibition of NF*κ*-B and IFN*γ* signalling pathways and an enhancement of peroxisome proliferator-activated receptor *γ* (PPAR*γ*) expression and activity, leading to the modulation of apoptosis and differentiation [120, 121]. Moreover, it was reported that HDAC inhibitors including butyrate induce autophagy which shares some common signalling pathways and is mutually regulated with apoptosis in colon cancer cells.

The putative mechanisms responsible for the different response to butyrate in normal, IBD- and tumour-derived colon cells may include changes in butyrate transport and uptake, mainly due to a different expression of sodium-coupled monocarboxylate transporters [122] and the specific G-protein coupled receptor 43 [123]. Butyrate metabolism is impaired in intestinal inflamed mucosa of patients with IBD. Disturbances in butyrate oxidation, the balance between butyrate and glucose oxidation, ROS generation in mitochondria, and differences in the overall cellular context play a role, too [124, 125].

## 5. Interactive Effects of Dietary Fatty Acids with TNF Family Cytokines

**Figure 1:**
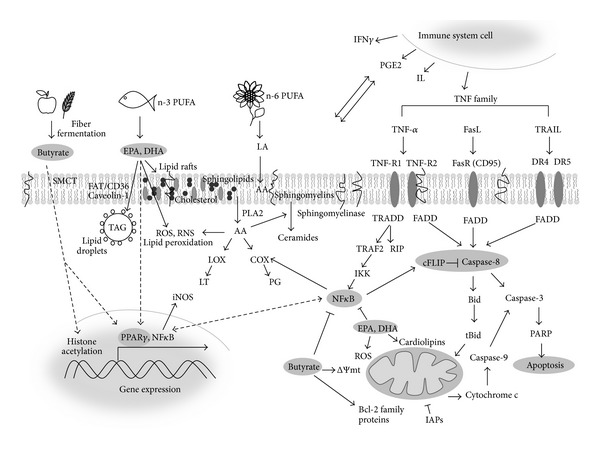
Schematic simplified demonstration of the main mechanisms supposed to be involved in fatty acid and TNF family interaction at different levels of colon epithelial cell organisation. Fibre fermentation product butyrate is transported by SMCT into the cells, where it may inhibit histone deacetylation, activate transcription factor PPAR*γ*, inhibit NF*κ*B, and affect ΔΨmt and expression and balance of pro- and antiapoptotic members of Bcl-2 family proteins. Dietary PUFAs of the n-6 (LA, AA-plant oils) and n-3 series and (EPA and DHA, fish and algal oils) transported into the cells by FAT/CD36 are stored in lipid droplets in TAG form or incorporated into plasma and mitochondrial membrane lipids thus modulating composition and structure of lipid rafts and caveolae. PUFAs are released from membrane phospholipids by PLA2 and metabolized by COX and LOX enzymes to various types of products such as LTs and PGs. PUFAs can also modulate ceramide production and their metabolism is source of ROS and RNS and induces lipid peroxidation. Incorporation of PUFAs into mitochondrial cardiolipins influences mitochondrial metabolism and induction of apoptosis. EPA and DHA activate PPAR*γ* and inhibit NF*κ*B, thus suppressing iNOS expression and RNS production. TNF family cytokines TNF-*α*, TRAIL, and FasL bind to their receptors TNFR1 and 2, DR4 and DR5, or FasR (CD95), respectively. Programmed cell death is regulated by formation of DISC consisting of FADD and procaspase-8, whose activation may be inhibited by cFLIP. Cleavage of Bid protein to tBid mediates connection between extrinsic and intrinsic (mitochondrial) apoptotic pathway. Changes in mitochondria (involving cardiolipin modulation), mitochondrial transition pore opening, decrease of ΔΨmt, and production of ROS influence the activation of pro- and antiapoptotic proteins of the Bcl-2 family and release of proapoptotic proteins (e.g., cytochrome c) into the cytosol. Subsequent activation of caspase-9 and caspase-3, cleavage of PARP, and execution of apoptosis can be inhibited by IAPs. TNF-*α* activates NF*κ*B via pathway involving TRADD, RIP, TRAF2, and IKK proteins. The interaction between epithelial cells and cells of immune system, which are the source of inflammatory mediators (IFN*γ*, IL, and PGE2), is also indicated. The dashed arrows-simplified signalling pathways. AA: arachidonic acid; cFLIP: FLICE-like inhibitory protein; COX: cyclooxygenase; ΔΨmt: mitochondrial membrane potential; DHA: docosahexaenoic acid; DISC: death-inducing signaling complex; DR: death receptor; EPA: eicosapentaenoic acid; FADD: Fas-associated DD protein; FasL: Fas ligand; FasR: Fas receptor; FAT/CD36: fatty acid translocase; IAPs: inhibitor of apoptosis proteins; IKK: inhibitor of NF*κ*B kinase; IFN*γ*: interferon*γ*; IL: interleukin; iNOS: inducible nitric oxide synthase; LA: linoleic acid; LOX: lipoxygenase; LT: leukotrienes; NF-*κ*B: nuclear factor *κ*B; PARP: poly-ADP ribose polymerase; PG: prostaglandins; PGE2: prostaglandin E2; PLA2: phospholipase A2; PPAR*γ*: peroxisome proliferator-activated receptor *γ*; PUFA: polyunsaturated fatty acids; RIP: receptor-interacting protein; RNS: reactive nitrogen species; ROS: reactive oxygen species; SMCT: sodium-coupled monocarboxylate transporters; TNF: tumor necrosis factor; TNFR: tumor necrosis factor receptor; TRADD: TNFR1-associated death domain protein; TRAF2: TNF receptor-associated factor 2; TRAIL: tumour necrosis factor-related apoptosis-inducing ligand.

It is suggested by us and others that dietary fatty acids (such as essential PUFAs or butyrate) and endogenous regulators from the TNF family can mutually interact and thus modulate the behaviour of colon epithelial cells. Such interactions may result in an altered production and activity of proinflammatory TNF family cytokines or an enhancement of their antiproliferative and proapoptotic effects. Moreover, these effects may be different in normal and cancer cells. Therefore, in the following sections we summarise the knowledge about these interactions, their possible mechanisms, and outcomes for intestinal cell behaviour and pathologies. The possible application of such knowledge for the prevention and therapy of colon inflammation and cancer is also outlined. Main mechanisms supposed to play the role in the fatty acid and TNF family cytokine interaction are schematically presented in Figure 1.

### 5.1. Interaction of TNF Family Cytokines with PUFAs

The effects of TNF family molecules on cancer cells could be significantly modulated by PUFAs and their metabolites. Previously we evidenced the potentiating effects of various inhibitors of AA metabolism on TNF-*α* induced apoptosis and differentiation in human leukaemic cell lines [126–128]. This phenomenon was also verified in human colon epithelial cells [129]. Our further results showed that the pretreatment of human colon cancer HT-29 cells with low doses of AA or DHA may prepare a permissive environment for a more effective apoptotic action of TNF family molecules. Importantly, low concentrations of both AA and DHA increased the apoptosis induced by anti-Fas antibody or TNF-*α*, which involved enhanced ROS production and decrease of mitochondrial membrane potential and caspase activation. Compared to AA, DHA showed more pronounced effects in lower concentration [130]. Similarly, the cotreatment of HT-29 cells with DHA enhanced TRAIL-induced apoptosis supporting the mitochondrial intrinsic pathway [131]. Recently, we confirmed these effects using several other colon cell lines (including TRAIL-resistant SW620 cells), which were pretreated with DHA. We clarified the key mechanisms of DHA and TRAIL interaction including the role of mitochondria, specific lipids, and signalling pathways (submitted manuscript). These results are promising, because in spite of the fact that TRAIL may be a selective anticancer agent, many cancer cells are resistant to its effects. This resistance may occur at different levels of intracellular signalling pathways, and manipulation of their individual components such as downregulation of antiapoptotic molecules or upregulation of proapoptotic factors can change the threshold for apoptosis induction by this cytokine [55]. This can be right achieved by combined treatments of TRAIL with selected agents specifically targeting the abovementioned molecules, such as chemotherapeutic DNA damaging drugs [53, 132] or fatty acids [131].

Summarising our results we have recognised that the cell response to molecules regulating cytokinetics as well as to lipid compounds is cell type-specific and may depend on the cell genetic background and the level of transformation. In colon cell lines derived from nontumour tissues or tissues on various stages of malignancy we detected a different sensitivity to TNF family cytokines, PUFAs as well as butyrate [65, 133, 134]. Generally, investigation of the molecular mechanisms and cellular specificity of PUFAs as well as TNF family cytokines enables us to determine also the possible mechanisms of their interaction.

PUFAs are actively transported into the cells by fatty acid translocase (FAT/CD36), and their incorporation alters the cellular lipid composition and fatty acid spectrum and shifts redox balance and the formation of various products of lipid metabolism [98]. In response to the elevated fatty acid content, complex and metabolically active organelles called lipid droplets (LDs) are formed. LDs are fundamental components of intracellular lipid homeostasis because they play a role in lipogenesis and lipolysis, serve as an important reservoir of signal molecules, and appear to be directly involved in membrane traffic and phospholipid recycling [135].

DHA, the longest (22 carbons) and the most unsaturated (6 double bonds) PUFA, is rapidly incorporated into the plasma as well as mitochondrial membrane phospholipids and also induces LD formation. It was shown to significantly alter the lipid microdomain (lipid rafts and caveolae) composition and the properties which increase or decrease specific receptors in lipid rafts accompanied by altered phosphorylation and thus activation of receptor and associated signalling kinases [136–138]. For example, after changes in membrane microdomains upon DHA treatment, epidermal growth factor receptor is excluded from caveolin-rich membrane fractions resulting in the subsequent downregulation of ERK signalling in three different cancer models [139]. The position and activity of TNFR, Fas, or DRs may be influenced in a similar way. Our results evidenced that increased DR5 surface expression, relocalisation of DRs to lipid rafts, and accelerated TRAIL internalisation are important for the sensitisation of colon and prostate cancer cells to TRAIL-induced apoptosis by platinum complexes [132]. Attention should be paid when applying TRAIL in combination with other drugs known for their ability to increase the DR surface level in cancer cells, as nonapoptotic signalling might also be increased under some circumstances.

DHA is specifically incorporated into the mitochondrial tetra-acyl phospholipid, cardiolipin (CL). Altered CL unsaturation and oxidative susceptibility of the mitochondrial membrane modulate the binding and activities of associated proteins, mainly of the pro- and antiapoptotic proteins of the Bcl-2 family. Changes of mitochondrial transition pore opening, decreased mitochondrial membrane potential, CL oxidation, and the release of proapoptotic cytochrome c and Apaf1 molecules are necessary for the execution of the intrinsic mitochondrial apoptotic pathway [140, 141]. Thus, in colonocytes and colon cancer cells, DHA alone or in combination with other agents can induce apoptosis by promotion of the mitochondrial apoptotic pathway as reported by us and others [131, 133, 142].

The beneficial effects of n-3 PUFA are of particular interest because IBD patients are very sensitive to nutraceutical approaches [143]. Using a fat-1 transgenic mice model, an inhibitory effect of endogenously synthesised n-3 PUFA on COX-2 gene expression was demonstrated during acute and chronic colitis accompanied by marked reduction in proinflammatory interleukins such as IL-1*α*, IL-6, and IL-18 and molecules endowed with chemotactic activity for granulocytes [144]. Moreover, endogenously synthesised n-3 PUFAs in such transgenic animals prevent colon cancer development by several mechanisms [145]. The networking of pathways of eicosanoid formation and inflammatory cytokine production represents a very important issue, but it concerns mainly the interaction of immune cells with other cell types and is not discussed in this review [146, 147].

An important consequence of PUFAs and TNF family cytokines interaction is represented by the modulation of proliferation and mainly cell death of cancer cells. Among other mechanisms, the participation of ceramides, which were already shown to play an important role in programmed cell death of cancer cells, was demonstrated [148]. Free AA has been shown to be an important mediator of TNF-*α* induced apoptosis via activation of sphingomyelinase and formation of ceramides [149]. TNF-*α*-mediated cell killing was inhibited by an increased AA metabolisation by COX-2 overexpression [150] or by blocking endogenous AA release in a mutant cell line with reduced cPLA2 activity [151]. The role of ceramide in Fas-mediated apoptosis has also been well documented [152]. Ceramide enables the Fas receptor to cluster to increase Fas-mediated apoptosis [153] and to modulate Fas receptor activation [72]. Authors identified XIAP and cIAP1 as molecular targets of ceramide.

The higher expression and secretion of various proinflammatory cytokines and their autocrine and paracrine functions play an important role in the activation of transcription factors, which in turn promote tumorigenesis. NF-*κ*B serves as a vital biomolecule that transcribes a number of proinflammatory cytokines and antiapoptotic proteins. NF-*κ*B is also known to play a critical role in the regulation of the inducible nitric oxide synthase (iNOS) gene. iNOS is an enzyme dominantly expressed during inflammatory reactions, and high amounts of nitric oxide (NO) have been demonstrated in pathophysiological processes, such as acute or chronic inflammation and tumorigenesis. Proinflammatory cytokines can also activate Jak3/Stat3 signalling pathways, thereby increasing inflammation and cell survival. The expression of IL-1*β*, IL-2, IL-4, IFN*γ*, TNF-*α*, iNOS, COX-2, Jak3, Stat3, and NF-*κ*B was increased in the early stages of experimental CRC. N-3 PUFAs suppress the activity of NF-*κ*B and thus reduce the production of proinflammatory enzymes and cytokines, including COX-2, TNF-*α*, and IL-1*β* [102]. The protective role of n-3 PUFAs, which suppressed the activity of NF-*κ*B and iNOS and increased the expression of transforming growth factor *β*, thus preventing colitis and CRC, was confirmed using fat-1 transgene mice [154].

*In vitro* studies have shown that n-3 PUFAs inhibit cell proliferation and induce apoptosis in cancer cells through the activation of transcription factors PPARs [155]. They influence lipid homeostasis and may be involved in the regulation of cell differentiation and death. The differential activation of PPAR isoforms (*α*, *β*/*δ*, *γ*) and PPAR-regulated genes by specific dietary fatty acids may be central to their distinct roles in cancer [156]. The data from a human case-control study suggest that PPAR*γ* may be associated with many aspects of CRC including insulin- and inflammation-related mechanisms [157]. In CRC patients, adipocyte dysfunctions creating a proinflammatory environment with upregulated STAT3 and the concomitant decrease of PPAR*γ* and adiponectin in white adipose tissue were detected with respect to healthy subjects. DHA was shown to have protective effects reestablishing the equilibrium between STAT3 and PPAR*γ* [87].

Wnt/*β*-catenin pathway is constitutively activated in more than 90% of human CRC. The activated *β*-catenin stimulates cell proliferation and survival; however, its antiapoptotic mechanisms are not fully understood. Recently, using a mouse model of inflammation-associated CRC and human colon cancer HCT-116 cells, Han et al. evidenced that the resistance of colon cancer cells to apoptotic effects of TNF-*α* is mediated by the activated nuclear *β*-catenin which blocks caspase cleavage of retinoblastoma protein. Further, the activated *β*-catenin can facilitate endosomal trafficking of internalised TNF-*α* to suppress caspase-8 activation in colon cancer cells [158]. Since DHA was shown to inhibit the Wnt pathway and activation of *β*-catenin, this mechanism may also play a role in its apoptosis-supporting effects [159].

### 5.2. Interaction of TNF Family Cytokines with Butyrate

Butyrate has been shown to have protective effects on inflammatory diseases such as UC and inflammation-mediated CRC. The ability of butyrate to trigger cancer cell apoptosis is one of the main features of its anticancer activity [160]. It induces apoptosis via mitochondria, efficiently modulating the level of Bcl-2 family and activating of caspases. Upregulation of the proapoptotic Bak and Bax, downregulation of the antiapoptotic Bcl-x_L_, XIAP, and survivin, or activation of caspase-3 and Bid after butyrate treatment were detected [161–163]. Butyrate was also shown to effectively modulate the extrinsic apoptotic pathway and to affect the initial steps of DR-mediated apoptosis at the level of DR, FADD, or cFLIP protein [49]. By affecting the level of these proteins, it could effectively contribute to the enhancement of apoptosis induced by TNF-*α*, TRAIL, or FasL [164].

Using various types of colon cancer cell lines, it was reported by us and others that the dual treatment with TNF-*α* and butyrate significantly increased apoptosis and decreased the differentiation induced by butyrate in HT-29 cells [165, 166]. Our experiments also documented that the sensitivity of colonic cells to TNF-*α* and various inhibitors of AA metabolism is dependent on the differentiation status. The apoptotic effect of dual treatment with butyrate and TNF-*α* can be potentiated by several different COX and LOX inhibitors [129].

The butyrate-mediated facilitation of the TNF-*α* induced death signal was also detected in COLO 205 adenocarcinoma cells, highly resistant to extrinsic apoptosis induced by death ligands [167, 168]. The immune escape of COLO 205 cells from TNF-*α* mediated apoptosis is probably caused by extensive shedding of TNFR1 and TNFR2 [169] and by a cFLIP protein inhibitory effect on caspase-8 activation [170]. The reduction of cFLIP protein and elevated TNFR1 expression after butyrate treatment corresponded with the higher sensitivity of COLO 205 cells to TNF-*α* induced apoptosis [171].

It could be supposed that butyrate and TNF-*α* might share similar signalling pathways including phospholipase C and protein kinase C [172, 173]. Butyrate-mediated changes of colon cancer cell sensitivity to TNF-*α* have been associated with the modulation of NF-*κ*B activity, which had a significant impact on proliferation, apoptosis, and inflammation in colon cancer cells. It was demonstrated that butyrate pretreatment of HT-29, SW480, and SW620 cells inhibits the TNF-*α* mediated p65 and p50 translocation to the nucleus, probably by suppressing the cellular proteasome activity and subsequently I*κ*B degradation through butyrate ability to inhibit HDAC [174–176]. Moreover, butyrate reduced inflammation in experimental colitis in rats [177] and decreased proinflammatory cytokine expression in intestinal biopsies from Crohn's disease patients via NF-*κ*B inactivation [178].

Butyrate pretreatment can lead to a significant enhancement of TRAIL-induced apoptosis in TRAIL-resistant cells [160]. Enhancement of apoptosis after combined treatment with butyrate and TRAIL was described in an HCT-116 colon cancer cell line. Butyrate caused a downregulation of XIAP protein and an upregulation of DR5, but no changes were detected on DR4 levels. Increased levels of DR5 were initiated by the binding of transcriptional factor Sp1 to* DR5* promoter after butyrate treatment [179]. Recently, an increased toxicity against gastrointestinal tumour cells* in vitro* and* in vivo* by combined treatment with sorafenib, HDAC inhibitors, and TRAIL was reported. The enhanced TRAIL cell killing correlated with the reduced Akt, ERK 1/2, and mTOR (mammalian target of rapamycin) activity and the enhanced cleavage of caspase-3 and reduced expression of Mcl-1 and Bcl-X_L_ [180].

Increased apoptosis in the combined treatment of butyrate and TRAIL was studied not only in colon cancer but also in other model systems including breast, bladder, and nervous cancer. Although individual mechanisms are cell type-dependent, several molecules (DR5, DISC, caspase-8, and Bcl-2 family proteins) play a crucial role across the models. Moreover, the combined treatment was not harmful to normal cells of different tissues, compared with their cancer counterparts [181, 182]. In addition, butyrate was reported to sensitise colon cancer cells to Fas-mediated apoptosis, mainly due to increased ROS production [165, 183].

Other data showed that inflammation can be affected both on the level of the immune system and colon epithelial cell interaction. In patients with UC it was discovered that butyrate inhibited the binding of HDAC1 to Fas promoter, which was hyperacetylated and led to an upregulation of Fas in T cells. Therefore, butyrate eliminated the source of inflammation by induction of T-cell apoptosis. Furthermore, butyrate suppressed IFN-*γ*-mediated inflammation in colon epithelial cells by preventing STAT-1 activation and blocking iNOS induction [69].

## 6. Importance of Fatty Acids for the Prevention and Therapy of Colon Inflammation and Cancer

Recently, it has been clear that dietary fatty acids not only serve as an energy source but also represent components which significantly influence physiological and pathophysiological processes from molecular and cellular to organismal levels. These compounds have also been considered as pharmaceutical agents which may have beneficial or detrimental impact on the body and may influence the effects of other drugs [108, 184]. The research concerning PUFAs as well as butyrate is closely related to the prevention and therapy of serious human diseases including cancer and interferes with other fields such as pharmacology and nutritional and health policy. Therefore, there is more need for a critical global overview and a consideration of the future perspectives [83, 185].

There are many reports showing the advantage of n-3 PUFAs as components of clinical enteral and parenteral lipid emulsions. Compared to the widely used emulsions from soybean oil containing mostly n-6 linoleic acid, other types with fish oil containing n-3 EPA and DHA were reported to have biological activities beneficial to patients [186, 187]. Many excellent reviews from experimental and clinical studies have been published on this topic, and thus we only summarise that nutritional supplementation with n-3 PUFA may influence inflammation, susceptibility to infection, and immune cell function and thus may affect immunological response, cachexia, and exert beneficial effects on the whole organism, which may then fight better against inflammation and cancer [188–191]. Besides fish oil, algal oil seems to be a better source of n-3 PUFAs due to gastrointestinal complaints of fish oil, especially in high-dose therapy [192]. To refine recommendations for the intake of individual n-3 PUFAs, differential effects of EPA, DHA, and their metabolites may be taken into account. The divergent incorporation into individual cellular lipids, activation of the signalling pathways and transcription factors, or the potency of their metabolites can contribute to diversity in the cellular response [193].

The importance of n-3 PUFAs as well as butyrate in relation to TNF family cytokines lies particularly in their ability to decrease the production of these cytokines and other factors (eicosanoids and interleukins) in the organism, to interfere with their signalling pathways, and thus to prevent the inflammatory response. Supplementation with DHA and EPA allows using lower doses of corticoids in IBD therapy [194].

Cancer anorexia and cachexia are major factors contributing to the weakening of the already compromised immune system of cancer patients. There is evidence suggesting that elevated levels of proinflammatory cytokines are associated with cancer-related cachexia [195, 196]. The treatment with fish oil or NSAIDs was shown to attenuate systemic inflammation and improve cachexia because it decreased production of active eicosanoids as well as level of proinflammatory and procachectic cytokines [197]. 

An important aspect is represented by the reported DHA and/or butyrate ability to support apoptosis induced by TNF cytokines, which is promising for use as a supportive anticancer agent. The therapeutic use of the TNF-*α*/TNFR, the FasL/Fas, or TRAIL/DRs in cancer treatment has been hampered by severe side effects [33, 198]. The systemic administration of TNF-*α* causes a septic shock-like response possibly mediated by NF-*κ*B activation, and the injection of an agonist antibody to Fas can be lethal. Moreover, many cancer cells are resistant to apoptosis induced by these cytokines [43, 48]. Thus, particularly the ability of n-3 PUFAs to decrease the therapeutic doses, to overcome the resistance, or to improve the overall state of the organism could be considered. Moreover, the interaction with TRAIL overcoming cancer cell resistance and being selective for cancer cells is highly important. However, it is necessary to further investigate and verify these possibilities before future clinical use.

There are studies showing mainly preventive but also potential therapeutic applications of butyrate, which aim to exploit its ability to regulate inflammation, apoptosis, and ion uptake. Anti-inflammatory effects of butyrate were studied in patients with UC or Crohn's disease as well as on animal models, where in several studies it reduced the clinical and inflammatory index [121]. Moreover, its ability to support the effects of other anti-inflammatory and anticancer agents (such as NSAIDs and apoptotic cytokines) is promising for clinical use. The advantages of treatment with butyrate include practically no adverse side effects and its possible oral administration, although its unpleasant taste and odour make it extremely difficult [199].

## 7. Conclusion

From the data described above it appears that the targeted use of a specific type of fat and fibre (PUFAs and butyrate) may have a number of beneficial effects both in physiological and pathophysiological conditions particularly in the intestine. Both PUFAs and fibre (source of butyrate) are natural dietary components which represent a nontoxic way for positive modulation of intestinal cell physiology, improvement of inflammatory conditions and cancer treatment outcomes, and slowing down or preventing the recurrence of IBD or CRC. Important issue represents the ability of dietary fatty acids to modulate production and function of endogenous regulators from TNF family and thus positively or negatively influence the behaviour of intestinal cells. Understanding the molecular mechanisms of both PUFA and butyrate effects and their interaction with specific type of cytokines may help to optimise the composition of clinical nutrition and therapeutical approaches for patients with IBD and intestinal neoplasms. However, as with other agents, they have to be applied carefully, based on solid scientific evidence of their mechanisms of action from the molecular and cellular up to the organismal levels.

## Abbreviations

AA: Arachidonic acid
ALA: *α* Linolenic acid
APC: Adenomatous polyposis coli
cFLIP: FLICE-like inhibitory protein
CL: Cardiolipin
COX: Cyclooxygenase
CRC: Colorectal cancer
DD: Death domain
DHA: Docosahexaenoic acid
DISC: Death-inducing signaling complex
DR: Death receptor
EPA: Eicosapentaenoic acid
ERK: Extracellular signal-regulated kinases
FAP: Familial adenomatous polyposis
FADD: Fas-associated DD protein
FAT/CD36: Fatty acid translocase
HDACI: Histone deacetylase inhibitor
HIF-1*α*: Hypoxia-inducing factor 1*α*
IAPs: Inhibitor of apoptosis proteins
IBD: Inflammatory bowel disease
IL: Interleukin
iNOS: Inducible nitric oxide synthase
JAK: Janus kinase
IFN: Interferon
JNK: c-Jun N-terminal kinase
LA: Linoleic acid
LD: Lipid droplet
LOX: Lipoxygenase
mTOR: Mammalian target of rapamycin
NF-*κ*B: Nuclear factor *κ*B
NSAIDs: Nonsteroidal anti-inflammatory drugs
PGE2: Prostaglandin E2
PLA2: Phospholipase A2
PPAR*γ*: Peroxisome proliferator-activated receptor *γ*
PUFA: Polyunsaturated fatty acids
RIP: Receptor-interacting protein
RONS: Reactive oxygen and nitrogen species
SCFA: Short-chain fatty acid
STAT: Signal transducer and activator of transcription
TNF: Tumor necrosis factor
TRAIL: Tumour necrosis factor-related apoptosis-inducing ligand
UC: Ulcerative colitis.
